# Post‐translational modifications of histones: Mechanisms, biological functions, and therapeutic targets

**DOI:** 10.1002/mco2.292

**Published:** 2023-05-20

**Authors:** Ruiqi Liu, Jiajun Wu, Haiwei Guo, Weiping Yao, Shuang Li, Yanwei Lu, Yongshi Jia, Xiaodong Liang, Jianming Tang, Haibo Zhang

**Affiliations:** ^1^ Cancer Center Department of Radiation Oncology Zhejiang Provincial People's Hospital Affiliated People's Hospital Hangzhou Medical College Hangzhou Zhejiang China; ^2^ Graduate Department Bengbu Medical College, Bengbu Anhui China; ^3^ Otolaryngology & Head and Neck Center Cancer Center Department of Head and Neck Surgery Zhejiang Provincial People's Hospital Affiliated People's Hospital, Hangzhou Medical College Hangzhou Zhejiang China; ^4^ Graduate Department Jinzhou Medical University Jinzhou Liaoning China; ^5^ Department of Radiation Oncology The First Hospital of Lanzhou University Lanzhou University Lanzhou Gansu China

**Keywords:** acetylation, cancer, methylation, phosphorylation, post‐translational modifications

## Abstract

Histones are DNA‐binding basic proteins found in chromosomes. After the histone translation, its amino tail undergoes various modifications, such as methylation, acetylation, phosphorylation, ubiquitination, malonylation, propionylation, butyrylation, crotonylation, and lactylation, which together constitute the “histone code.” The relationship between their combination and biological function can be used as an important epigenetic marker. Methylation and demethylation of the same histone residue, acetylation and deacetylation, phosphorylation and dephosphorylation, and even methylation and acetylation between different histone residues cooperate or antagonize with each other, forming a complex network. Histone‐modifying enzymes, which cause numerous histone codes, have become a hot topic in the research on cancer therapeutic targets. Therefore, a thorough understanding of the role of histone post‐translational modifications (PTMs) in cell life activities is very important for preventing and treating human diseases. In this review, several most thoroughly studied and newly discovered histone PTMs are introduced. Furthermore, we focus on the histone‐modifying enzymes with carcinogenic potential, their abnormal modification sites in various tumors, and multiple essential molecular regulation mechanism. Finally, we summarize the missing areas of the current research and point out the direction of future research. We hope to provide a comprehensive understanding and promote further research in this field.

## Graphical abstract

Histone tails are subject to a variety of post‐translational modifications. We have introduced histone acetylation, methylation, phosphorylation, ubiquitination, malonylation, crotonylation, propionylation, butyrylation, and so forth. They participate in many life activities through different related histone sites.

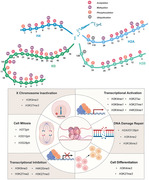

## 1 INTRODUCTION

**FIGURE 1:**
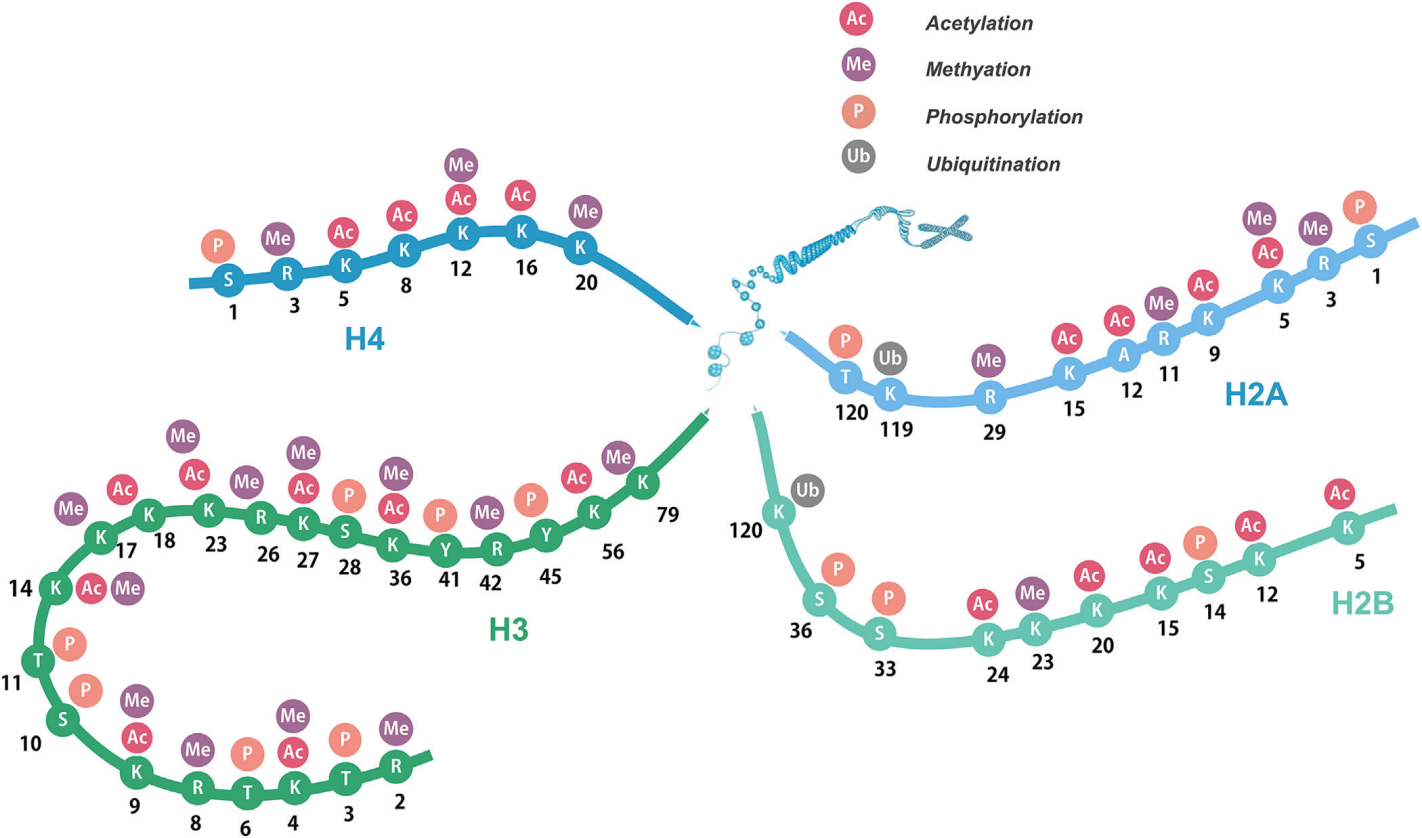
Post‐translational modifications (PTMs) of histone amino terminus. The DNA is wrapped in two circles, around the DNA and the histone octamer. Histones in nucleosomes (two each of H2A, H2B, H3, and H4). Histone tails are subject to various PTMs that affect not only the overall compression of chromatin but also gene expression. This diagram shows some of the modifications at specific residues: acetylation (Ac), methylation (Me), phosphorylation (P), and ubiquitination (Ub).

As a branch of genetics, epigenetics refers to the heritable gene expression changes caused by external modification without changing the DNA sequence. The main mechanisms involved are RNA interference, DNA methylation, histone modifications, and so on.^1^ Post‐translational modifications (PTMs) belong to the field of epigenetics. It does not change DNA sequence but can change expression and function levels, which provides a new explanation for many life phenomena. Histones can be modified in many ways, and the most common ones include methylation, acetylation, phosphorylation, and ubiquitination. However, with the development of high‐sensitivity mass spectrometry technology, various new histone acylation markers such as malonylation, crotonylation, propionylation, and butyrylation have been found in recent decades. In addition, histone lactylation, a new modification type discovered in 2019, is also a hot research topic at present.^2^, ^3^ These modifications are associated with the structural formation of activated or deactivated chromatin. Since most PTMs are reversible, the function of the proteome can be regulated in a cell‐type‐specific manner to describe gene expression. Histones H3 and H4 have a long tail protruding from a specific position in the nucleosome nucleus, which is covalently modified. The cores of histones H2A and H2B also underwent modifications. The diversified modification of the histone amino‐terminal expands the genetic code, so it is called the “Histone Code”^4^, ^5^ (Figure 1). Histone methylation modification is more stable than other PTMs, so it is the most suitable for stable epigenetic information. However, acetylation modification has high dynamics, and there are other unstable modification methods, such as ubiquitination, phosphorylation, and malonylation. These modifications affect chromatin more flexibly and exert its regulatory function through the combination of various modification methods. Acetylation was the first PTM to enter people's attention, so it has been studied most deeply.

Acetylation modifications modulate protein function through many mechanisms, including enzyme activity, protein translocation, and crosstalk with other PTMs.^6^, ^7^, ^8^, ^9^, ^10^ Histone acetyltransferase (HAT) also performs multiple functions in DNA replication, gene expression, and immune regulation.^11^, ^12^, ^13^, ^14^, ^15^ Histone Acetyltransferase Binding To ORC1 (HBO1) is a classical acetyltransferase. The differential relationship between acetyltransferase HBO1 and the BRPF and JADE subunits determines which histone tails are acetylated.^16^ BRPF2‐HBO1 preferentially catalyzes histone H3/H4 acetylation, whereas JADE‐HBO1 modifies histone H4. In general, HBO1 is responsible for histone H3K9/14 and H4K5/8/12 acetylation.^11^, ^17^, ^18^, ^19^, ^20^ The BRPF2 subunit interacts with the MYST domain of HBO1 and promotes the activation of histone H3K14 acetylation.^21^, ^22^ It was found that the effect of the BRPF2‐HBO1 complex was not limited to the acetylation of H3/H4 but could also effectively catalyze propionylation, butyrylation, and crotonylation of H3/H4.^17^ In addition, there is strong evidence that HBO1 exerts its functional role as an oncogene in many human cancers. Besides, several methyltransferases like SMYD3, EZH2, and SETDB1 are also abnormally expressed in common human cancer lines.^23^ Therefore, researchers have attached great importance to epigenetic modifiers. Histone deacetylase (HDAC) inhibitors (HDACIs) are the most studied drug in the current research. This paper also introduces the research status of inhibitors of HAT, histone methyltransferase (HMT), and histone demethylase (HDM) in various tumors.

**FIGURE 2:**
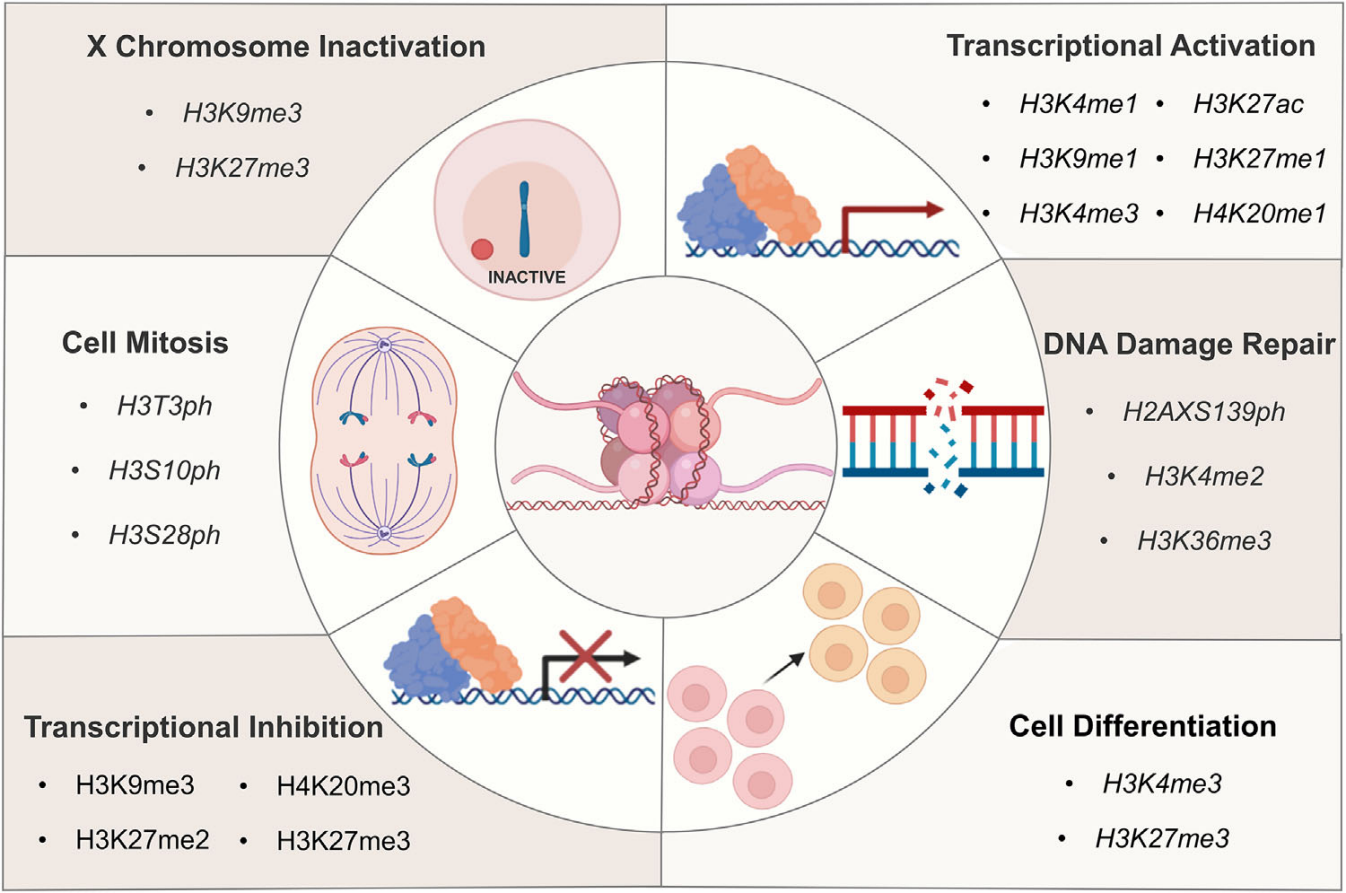
A wide range of biological effects mediated by histone modification.

Histone modification enzymes like HAT, HMT, HDAC and HDM's basic function is to regulate gene expression. For example, histone methylation often results in gene silencing, while demethylation is the opposite. Acetylation generally activates transcription, while deacetylation is the opposite. Many complex biological effects are produced on this basis. It is well known that histone codes can be involved in cell mitosis,^24^, ^25^, ^26^ DNA damage and repair,^27^, ^28^, ^29^ cell differentiation,^30^ X chromatin inactivation,^31^ and mediate transcriptional activation or inhibition,^32^, ^33^ leading to extensive biological effects (Figure 2). Histone modification can be understood as changes in enzyme activity. In the past decade, abnormal expression of histone modification enzymes has been detected in various tumors.^34^ Therefore, understanding the biological characteristics of PTMs is essential for understanding their pathophysiology. Histone modifications regulate gene expression by regulating enzyme activity, which is very similar to the nature of some therapeutic drugs that achieve therapeutic purposes by regulating enzyme activity. Thus, the biological study of histones is very promising for solving physiological and pathological problems and providing new disease prevention and treatment strategies. In this review, we focus on the basic regulatory rules of PTMs and their various biological functions in life activities and introduce the mechanism of abnormal histone modification behavior in various cancers. Second, we highlight the development status of therapeutic drugs for different abnormal histone‐modifying enzymes reported at present. In the end, we summarized the shortcomings of current research and potential research directions in the future.

## 2 PTMs OF HISTONES

There are many kinds of catalytic enzymes in cells that can chemically modify histones. Different forms of PTMs can weaken or strengthen the interaction between DNA and histones, resulting in gene activation or silencing. All these histone PTMs constitute the histone code, and the histone code determines the transcription status of local genomic regions. Therefore, studying various histone PTMs in a specific region or even the whole genome can reveal the active state of genes, which is very important for understanding genome programming in life activities.

### 2.1 Histone methylation

Histone methylation is an important modification that can change the structure of chromosomes. Twenty‐four methylation sites were identified, of which 17 were located in lysine and seven in arginine. Methylation is mainly catalyzed by HMT, which can be divided into histone lysine methyltransferase (KMT) and protein arginine methyltransferase (PRMT).^35^, ^36^, ^37^, ^38^ In addition, KMTs can be divided into SET‐containing and non‐SET domains according to the catalytic domain sequence. SET domain is an important domain of HMT, including SUV39H1/2, G9a, GLP, SMYD, SETDB1, EZH2 and others.^39^ However, a few proteins did not have the SET domain, except for the DOT1L protein.^40^ Mammalian PRMTs fall into two categories: the first includes PRMTs 1, 3, 4, 6, and 8, which catalyze the process of monomethyl arginine and asymmetric monomethyl arginine. The second class includes PRMT5 and PRMT7, which catalyze the process of monomethyl arginine and symmetric bis‐methylarginine.^41^ In addition, PRMT2 was confirmed due to its homology with known arginine methyltransferase enzymes, but its enzyme activity has yet to be determined. Many different methylation modification patterns can be evolved from different sites and histone methylation patterns, increasing the complexity and diversity of gene expression. Methylation modifications are also reversible. Two evolutionarily conservative HDM families have been identified: Jumonji C (JMJC) and lysine‐specific demethylase (LSD) protein families.^42^ LSD1 (KDM1A) is the first reported histone lysine demethylase (KDM). It catalyzes the demethylation of H3K4me1/2 and H3K9me1/2, specifically removes monomethyl and dimethyl labels from H3K4, and plays a vital role in carcinogenesis.^43^ Besides, LSD1 can also demethylate non‐histone proteins.^44^, ^45^

HMTs and HDMs carefully balanced histone methylation levels, making it easy to understand the close relationship between their disorders and cancers. There is evidence that abnormal histone methylation may be involved in some cancers.^46^, ^47^, ^48^ For example, the abundance of methyltransferase SMYD3 in normal human tissues is low, while it is highly expressed in liver cancer, lung cancer, and pancreatic cancer.^49^ In addition, many other KMTs, such as EZH2, SETDB1, and DOT1 were also found to be related to the malignant progression of tumors. The researchers in developing HMT and HDM inhibitors have succeeded greatly. Effective inhibitors of various HMT and HDM are in different clinical stages or preclinical research stages. This will provide new ideas for the treatment of some tumors.

### 2.2 Histone acetylation

**FIGURE 3:**
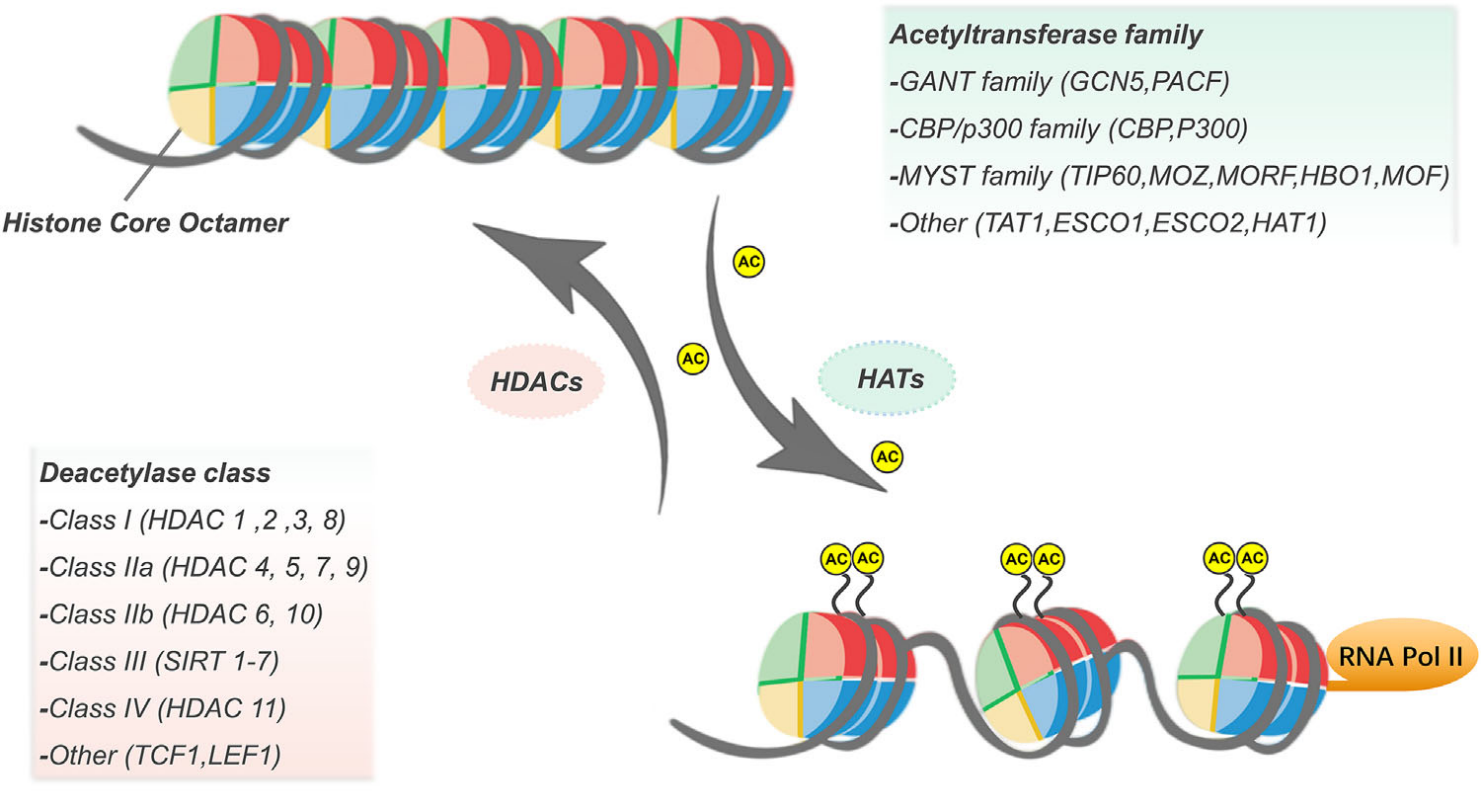
Histone acetylation levels are co‐regulated by HDACs (erasers) and HATs (writers). HATs are relatively distinct from each other. HDACs can be further classified as Class I, IIa, IIb, III, and IV.

Acetylation modifications are evolutionarily conserved and reversible PTMs and are among the first described histone modifications, with lysine acetylation modifications first identified in histones by Allfrey et al. (1964).^50^, ^51^ In the mammalian nucleus, HAT regulates histone acetylation while HDAC regulates deacetylation, thus maintaining a dynamic balance (Figure 3). HAT transfers “acetyl‐CoA,” promoting the relaxation of nucleosome structure and thus activating transcriptional activity. If HATs are inhibited, damaged DNA may not be repaired, leading to cell death. Thirteen HATs have been identified, and the HATs consist of three main families: CBP/p300, GCN5, and the MYST acetyltransferase family.^52^, ^53^ In contrast, HDACs deacetylate histones attach strongly to negatively charged DNA, convolute chromatin densely, and repress gene transcription. The human genome contains 18 HDACs. HDAC proteins are also known as lysine deacetylases (KDACs). HDACs (except class III HDACs) contain zinc and are known to be zinc^2^+‐dependent HDACs.^53^, ^54^, ^55^ They have classical arginase folding, structurally and mechanically different from sirtuins (class III), which fold into a Rossmann structure and are NAD‐dependent.^56^, ^57^ KDAC leads to histone deacetylation, a hallmark of gene silencing.^58^, ^59^, ^60^ Therefore, abnormal hyperacetylation or deacetylation can cause various diseases.^61^, ^62^, ^63^ Acetylation can weaken the binding of histone to negatively charged DNA.^64^, ^65^ This reduced combination allows chromatin amplification and genetic transcription. HDACs remove these acetyl groups, promote high‐affinity binding between DNA and histones, and prevent their transcription. This process is the typical mechanism of action of HDAC inhibitors (HDACIs). HDACs play key roles in many life activities, including gene expression regulation, cellular metabolism, chromatin remodeling, cancer, and aging.^66^, ^67^, ^68^, ^69^, ^70^

Epigenetic alterations are the most common abnormalities in cancer because of hundreds of potential targets.^71^, ^72^ The ability of HATs to manipulate chromatin structure and epigenetic framework is imperative for cell maintenance and survival.^73^, ^74^, ^75^ It plays multiple functional roles in regulating proliferation, apoptosis, epithelial mesenchymal transition (EMT), and CSC in a variety of human cancers.^76^, ^77^, ^78^, ^79^ To classify the functions of hundreds of proteins that regulate chromatin, enzymes that regulate PTMs are subdivided into “writers,” “erasers,” “readers,” and “movers.”^76^ The identification of mammalian HATs (writers) and HDACs (erasers), bromodomain‐containing domains (readers), and HDACIs have laid the foundation for in‐depth studies of histone acetylation modifications.^80^, ^81^ The interaction between HATs and HDACs affects homeostasis in the body, and they coordinate gene expression and metabolic processes.^69^ HATs and HDACs have important functional roles in some genetic diseases and human cancers, and their potential in treatment has attracted much attention. HDACIs lay a foundation for further study on the treatment method of histone acetylation modification. Currently, Food and Drug Administration (FDA)‐approved HDACI such as SAHA has been used clinically. In addition, clinical trials of several kinds of HDACIs, including entinostat, resminostat, and abexinostat, are also at the forefront of the present study.

### 2.3 Histone phosphorylation

Histone phosphorylation also belongs to the field of epigenetics, and its modification sites are serine, threonine, and lysine with a hydroxyl group. The process of histone phosphorylation leads to the decrease of affinity between histone and DNA. The loose chromatin structure was conducive to binding transcription factors to DNA. As a histone marker, phosphorylation modification is recognized and interpreted by effector proteins to change the structure of chromatin. Phosphorylated histone can be hydrolyzed to remove phosphate under the action of protein phosphatase. It can destroy the connection between histone and DNA, resulting in DNA damage.^82^, ^83^, ^84^ Histone phosphorylation is essential for cell division processes, which promote the agglutination of chromatin into chromosomes.^82^, ^85^ Histone phosphorylation participates in numerous cellular processes. Unlike acetylation and methylation, histone phosphorylation establishes interactions between other histone PTMs and acts as a platform that causes downstream cascading events.^86^ Similar to other PTM, histone phosphorylation is also reversible.^87^ With the deepening of research on histone phosphorylation, more and more discoveries have been made in this field, which have guiding significance for the research direction of many important life processes of cells.

Phosphorylation can occur in all core histones, and the phosphorylation of each core histone has different functions.^88^, ^89^, ^90^, ^91^, ^92^ DNA double‐strand break (DSB) can rapidly initiate H2AX phosphorylation at serine 139 (H2AX139ph) to generate γ‐H2AX, which is one of the earliest events after DSB.^93^, ^94^ In addition, H2BS32 is associated with cell transformation.^95^ The phosphorylation sites of H3 mainly include serine at positions 10 and 28 and threonine at positions 3 and 11.^96^, ^97^ Most studies have focused on the phosphorylation of H3S10, which is highly conserved and catalyzed by the kinase haspin.^98^ H3S10ph is primarily involved in chromosome aggregation during mitosis and meiosis and is commonly used as a marker of cell mitosis.^99^, ^100^, ^101^ Furthermore, an increase in H3S10ph has been found in many cancer types, and a high abundance of H3S10ph has been associated with poor prognosis.^102^, ^103^ At present, the research on histone H4 phosphorylation mainly focuses on serine 1. H4S1ph can be considered an epigenetic marker of sperm maturation.^104^ Although many sites of histone phosphorylation have been found, there is no report on effective inhibitors of histone phosphorylation, which is a valuable field and deserves more research in the future.

### 2.4 Histone ubiquitination

The modification of histone ubiquitination has been discovered for a long time. The ubiquitination modification of histone H2A was found even earlier than the ubiquitination‐mediated protein degradation. Like other protein ubiquitination modification processes, histone ubiquitination also requires three types of enzyme catalysis: ubiquitin activator enzyme E1, ubiquitin‐binding enzyme estradiol (E2), and ubiquitin‐protein ligase E3.^105^ Histone ubiquitination does not participate in protein degradation but provides a marker for its role in DNA damage response, gene transcription, and other aspects.^106^ Deubiquitinating enzymes include the ubiquitin carboxyl c‐terminal de‐hydrolase family and ubiquitin‐specific processing protease family.^107^ H2A and H2B are the most ubiquitinated histones among all histones.^108^ H2A is the earliest identified substrate for ubiquitination modification, and 5%−15% of histone H2A can be ubiquitinated in higher eukaryotes.^109^ The single ubiquitinated histone H2A modification site is lysine 119 (H2AK119ub), catalyzed by the ubiquitin E3 ligase polycomb repressive complex 1 (PRC1) of histone H2A.^110^, ^111^ In addition, H2Aub takes part in various life activities, such as gene transcription and DNA damage repair,^112^, ^113^, ^114^ which are strictly and finely regulated. H2Aub promotes the combination of histone H1 and nucleosomes and hinders the stripping of DNA on nucleosomes to achieve nucleosome stabilization.^115^ The ubiquitination site of H2B is also located at a lysine residue, particularly lysine 120 in mammals (H2BK120ub) and lysine 123 in yeast (H2BK123ub).^116^ In vivo, H2BK123ub is catalyzed by the E2 transferase of Rad6 and the E3 ligase Bre1.^117^, ^118^ The ubiquitination of H2B (H2Bub) shows rapid enrichment at the DSB site, indicating that H2B plays a direct biochemical role in DSB repair.^119^ It is worth mentioning that the effect of H2Bub in DSB repair may be related to its crosstalk with methylation. Studies have found that H2BK123ub could promote the methylation of H3K4, H3K46, and H3K79, which are essential for DSB injury repair.^120^, ^121^, ^122^ In addition, mono‐ubiquitination of H2BK120 is involved in the crosstalk with H3K79 methylation. Moreover, Armache et al. reported that H4K16ac and H2Bub can simultaneously and synergistically regulate the enzymatic activity of DOT1.^123^ The methyltransferase activity of DOT1 can be regulated by H4K16ac, which is further enhanced by H2BUb, thus achieving the optimal catalytic rate of DOT1. When H2Bub is present, the nucleosomes with H4K16ac and H2Bub mediate the optimal localization of DOT1 on the nucleosome, increasing the probability of trimethylation. However, this kind of crosstalk between various histone modifications involving ubiquitination, methylation, and acetylation is very rare. Therefore, the regulation of complex histone modifications and the unique cascade reactions involved in this activity have great research value and therapeutic potential in the future.

In addition, abnormal histone ubiquitination patterns have been proven to exist in many cancer types. BMI1, the core of PRC1, which ubiquitinates lysine 119 at histone H2A (H2AK119ub1), is highly expressed in gastric cancer (GC), colon cancer (CRC), and breast cancer.^124^, ^125^, ^126^, ^127^ BMI1 could activate INK4A/ARF signaling pathway, which is mediated by H2AK119ub, thus maintaining the self‐renewal ability of leukemia stem cells (LSC).^128^, ^129^ These results suggest a pathogenic potential at high levels of H2AK119ub. In contrast, immunohistochemical (IHC) analysis shows that the deletion of H2Bub accelerates the process of tumor occurrence.^130^, ^131^, ^132^ Notably, the depletion of RNF20 and H2BK120ub greatly reduces the expression of p53. In contrast, the expression level of c‐Myc and c‐Fos are increased.^133^ Global loss of H2BK120ub has also been found in triple‐negative breast cancers (TNBC), GC, and CRC.^132^, ^134^, ^135^ However, IHC results from estrogen receptor (ER)‐positive breast cancer have indicated elevated levels of H2BK120ub.^136^ Therefore, whether targeting H2AK119ub or H2BK120ub will inhibit or promote the viability of cancer cells may be unpredictable, and it is necessary to analyze the individuation of various cancer subtypes.

### 2.5 Histone propionylation and butyrylation

Methylation and acetylation were once considered the core modifications of “histone code” until the discovery of propionylation and butyrylation greatly expanded people's cognition. Histone propionylation and butyrylation were first reported in histone H4 in HeLa cells.^137^ Subsequently, researchers have found multiple catalytic sites for lysine propionylation (Kpr) and butyrylation (Kbu) in yeast histones and evolutionarily conservative eukaryotes.^138^ The acyl sources for Kpr and Kbu are propionyl‐CoA and butyryl‐CoA respectively.^139^, ^140^, ^141^, ^142^ These acyl donors, all of which originate from the fatty acid metabolism pathway, suggest a correlation between cell metabolism and histone modification. H3K14 is the site of Kpr and Kbu in vivo, and HAT and HDAC can catalyze the addition and removal of propionyl and butyryl groups. Studies have confirmed that the H3K14pr and H3K14bu processes are related to HAT and preferentially distribute at the promoter of the active gene^143^ Members of the HAT family, such as p300, CBP, and HBO1 are all involved in the modification of Kpr and Kbu.^17^, ^137^ GNAT family members GCN5 and PCAF also participate in the above acylation modification.^144^, ^145^ In addition, Sirt1/2/3 of the HDAC class can remove the propionyl and butyryl groups.^146^ Major histocompatibility complex class I polypeptide‐related sequences A and B (MICA/B) are a type of stress protein widely expressed in solid tumors and blood tumors.^147^, ^148^ Propionate induces and increases Kpr and Kbu, which regulate the expression of MICA/B, suggesting that propionate produced by bacteria or in cell metabolism processes has significant immunoregulatory functions and can prevent cancer.^149^, ^150^ HDACIs, as unique anti‐tumor drugs, can induce histone acetylation and the upregulation of Kbu in tumor cells. SAHA, a representative drug of HDACIs, can significantly induce multiple butyrylation sites, providing a new approach for the development of protein omics changes in neuroblastoma.^151^ However, this function of HDACIs in regulating Kbu has not been widely reported, and it is still unknown if there are other kinds of HDACIs with similar functions. In the future, whether SAHA can also regulate Kbu in other cancers and whether different kinds of HDACI can also regulate Kbu will be the focus of research.

### 2.6 Histone malonylation

Histone malonylation is the process of covalently binding malonyl groups to histone lysine residues under the catalysis of enzymes. Lysine malonylation (Kma) is a novel histone PTM first identified and reported by Chao et al. in 2011.^152^ This was immediately followed by Xie et al. identifying the Kma locus in yeast and HeLa cells.^153^ As a newly discovered form of acylation modification, malonylation takes part in the metabolic process, especially in energy metabolism. Du et al. detected high levels of Kma in mice with Type 2 diabetes, suggesting a potential pathogenic effect of abnormal Kma levels.^154^ Previous studies have confirmed that Sirt5 of class III HDACs can reverse the Kma reaction.^155^, ^156^ Currently, the role of Sirt5 as a de‐malonylase has been found; however, there is no relevant report on transferases that mediate malonylation. Our understanding of malonylation needs to be improved. Therefore, the study of the enzymes that catalyze the formation of malonylation is a potential research focus in the future, and the exploration of the relationship between malonylation and mammalian pathophysiology also involves extensive research content.

### 2.7 Histone crotonylation

Lysine crotonylation (Kcr) refers to a modification in which crotonoyl is transferred to lysine residue by histone crotonyltransferase (HCT). Kcr is distributed in core histones and some non‐histones. Zhao et al. first found the crotonic acylation modification in 2011 and defined it as a novel histone acylation modification.^157^, ^158^ Like other types of PTMs, crotonylation is also reversible and regulates gene expression. Crotonylation can be reversibly catalyzed by HCT and histone decrotonylase (HDCR). In addition, HATs have also been shown to have HCT activity, and HDACs were reported to have HDCR activity.^159^, ^160^, ^161^ The Taf14 protein containing the YEATS domain is a specific presence as a histone crotonylation modification “reader,”^162^, ^163^ laying a foundation for elucidating the biological function of Kcr. Moreover, Kcr is involved in spermatogenesis, gene transcription, and cancer occurrence.^164^ Male mouse germ cells have a high abundance of Kcr on sex chromosomes at anaphase, suggesting the importance of Kcr in spermatogenesis.^165^ Compared with histone acetylation, crotonylation modification of histones has a stronger transcriptional activation function because it is mainly distributed in the promoter of the active gene or the potential enhancer region, thus regulating gene expression.^166^, ^167^, ^168^ Results analyzed by Wan et al. using IHC staining showed that Kcr levels were decreased in GC and HCC. However, it is increased in thyroid, lung, esophageal, and pancreatic cancers.^169^ Different biological behaviors and functions of Kcr in various cancers indicate its complex regulatory mechanism. Its carcinogenic or anti‐cancer effect may be related to different Kcr sites. However, research on Kcr is still very scarce, and it is necessary to clarify the relationship between Kcr at different sites and its mediated biological functions in the future.

### 2.8 Histone lactylation

Histone lactylation was first reported by Zhao et al. in 2019, and it is one of the newly discovered histone PTMs.^3^ Lactate is an intermediate product produced during the metabolism of glucose, which often exists in two isomers, L‐lactate and D‐lactate, and the main product of glycolysis is the L‐lactate. L‐lactate can transform into the L‐lactyl‐CoA, and the accumulation of L‐lactate promoted the histone modification of lactylation.^170^, ^171^ Therefore, the levels of lactyl‐CoA and acetyl‐CoA may reflect the levels of lactylation and acetylation of histones. It was first reported that there were 28 lactate sites in histones, which were reported by Zhao et al. Later, Meyenn et al. found that global H3K18 lactylation distribution marked the active promoter regions of some highly expressed genes, which was positively correlated with gene expression.^3^, ^172^ In recent years, the study of epigenetic modifications has gained significant attention, and histone lactylation has been discovered to play a crucial role in immune regulation, inflammation, cancer, and other diseases.^173^, ^174^, ^175^, ^176^

The p300 of HAT family mediates the lactylation modification of H3 and H4, while the deacetylases HDAC1‐3 and SIRT1‐3 have the de‐L‐lactylase enzyme activity.^171^ These results indicate that histone lactate is also a reversible process. Warburg effect, also known as aerobic glycolysis, is a metabolic phenomenon in which cancer cells preferentially convert glucose to lactate even in the presence of oxygen. This process provides energy for cancer cell proliferation but is not an efficient way to produce adenosine triphosphate (ATP). The increased reliance on glycolysis for energy production is a hallmark of many cancer types and is thought to contribute to abnormal tumor growth and development.^177^ Therefore, histone lactylation in tumors is considered to be very likely abnormal. Furthermore, Yu et al. reported that histone lactylation plays an important role in tumor development for the first time. The level of histone lactylation is increased in ocular melanoma, which promoted the expression of the oncogene, YTHDF2, thus recognizing and degrading m6A‐modified PER1 and TP53 mRNAs, leading to malignant progress of ocular melanoma and poor clinical prognosis.^178^ In addition, the increased level of histone lactylation in non‐small cell lung cancer (NSCLC) will lead to high levels of G6PD and SDHA and metabolic disorder.^179^ However, demethylzeylasteral, a small molecule drug, can significantly reduce the lactylation modification level of H3K9 and H3K56, thus inhibiting the self‐renewal and proliferation ability of liver cancer stem cells.^180^ In addition, Liu et al. reported that histone lactylation relies on the PDGFRβ pathway to enhance tumor cell proliferation and migration in clear cell renal cell carcinoma.^181^ Histone lactylation is closely related to various life activities in mammals and regulates the function of genes through multiple molecules. Existing results indicate that histone lactylation plays an important role in the occurrence and development of tumors, providing new ideas and directions for tumor treatment and intervention. Unfortunately, the understanding of histone lactylation is not deep enough. At present, only a few tumors have been found to be regulated by histone lactylation. Therefore, it is of great significance to study the function and regulation mechanism of histone lactylation in physiological and pathological processes for further understanding the disease occurrence process and future clinical application.

## 3 ABNORMAL EXPRESSION OF HISTONE‐MODIFYING ENZYMES AND HUMAN CANCERS

Histone‐modifying enzymes play a vital role in diverse DNA‐templated processes including gene expression, thus regulating cell growth and signaling pathways.^182^, ^183^ Acetylation and deacetylation in vivo are generally considered to be dynamically balanced, but under certain pathological conditions, such as genetic defects of metabolizing enzymes, accumulation of upstream metabolites and synergistic action of these metabolites with enzymes will result in an increase of corresponding PTM levels, leading to disease occurrence.^182^ In addition, histone modifications are also recognized as potential markers for the occurrence and development of many diseases, especially various cancers. As a multi‐factor disease, cancer has always been a difficult problem for people because of its remarkable complexity. It is worth noting that the abnormal regulation of histone PTMs is gradually considered an important marker of cancer. Abnormal histone modification patterns have been found in various human cancers (Table 1), which regulate the occurrence and development of cancers. We discuss the roles of different types of histone modifications and the corresponding catalytic enzymes in cancer biology and explore the possibility of using them as prognostic markers through the following 10 common cancers, which are most closely related to abnormal histone PTMs.

**TABLE 1:** Abnormal global histone modification pattern in cancer.

**Study**	**Cancer type**	**Relevant histone modifications**	**Ref**.
Sarkar et al. (2015)	Glioblastoma (GBM)	H3K27me3, H3K4me3, H3K9me3	^187^
Jang et al. (2012)	Lung cancer	H3K9ac, H3K9me3, H4K16ac	^198^
Rodriguez et al. (2007)	Lung cancer	H3K4me2, H3K9ac, H2AK5ac	^199^
Jin et al. (2014)	Hepatocellular carcinoma (HCC)	H3K27me3, H3K4me3	^222^
Cai et al. (2021)	HCC	H3K4me2, H4R3me2	^223^
Jang et al. (2008)	Gastric cancer (GC)	H3K9me3	^240^
Dawson et al. (2010)	Pancreatic cancer	H3K4me2, H3K9me2, H3K18ac	^251^
Edelweiss et al. (2016)	Pancreatic cancer	H4K12ac, H3K18ac	^252^
Ostrowski et al. (2014)	Colon cancer (CRC)	H3K27ac	^265^
Kuppen et al. (2014)	CRC	H3K4me3, H3K9me3, H4K20me3	^266^
Chapkin et al. (2017)	CRC	H3K4me3	^267^
Harithy et al. (2021)	CRC	H3K9me	^268^
Kuppen et al. (2015)	CRC	H4K16ac, H3K56ac	^269^
Ellis et al. (2009)	Breast cancer	H9K3ac, H18K4ac, H12K3ac, H4K2me4, H20K3me4, H3R2me2	^289^
Beyer et al. (2020)	Breast cancer	H3K4me3, H3K9ac	^294^
Robinson et al. (2022)	Ovarian cancer (OC)	H3K27me3	^314^
Huang et al. (2019)	Leukemia	H3K9me3	^346^

Abbreviations: ac, acetylation; me, methylation.

### 3.1 Glioblastoma (GBM)

GBM, a tumor originating from glial cells, is the most common primary intracranial tumor. Its prognosis is usually unsatisfactory even after appropriate surgical resection, radiotherapy, or chemotherapy.^184^, ^185^ K27M mutation was found in 30% of children with high‐grade GBM, which was related to adverse clinical outcomes.^186^, ^187^ The decrease of H3K4me3 content in the promoter was also detected.^188^ Christina et al. found that the changes in gene expression of various methyltransferases, acetyltransferases, and deacetylases promoted the pathogenesis of GBM.^189^ The nuclear expression levels of lysine methyltransferases SETDB1, KMT5B, Suv‐39h1, and EZH2 in GBM are up‐regulated, compared with normal tissues, and correlated with advanced histological grades.^190^, ^191^, ^192^, ^193^ Besides, the inhibition of LSD1 in GBM has been discovered by Chandra et al. to sensitize cells to HDACIs, so LSD1 and HDAC can work together to regulate cell death in GBM cell lines.^194^ Besides, patients with higher KDM5B levels in GBM usually have poorer overall survival rates and can promote cancer cell proliferation by regulating p21 expression.^195^ HAT HBO1 has also been confirmed to have malignant potential in the development of GBM. The mechanism of action of NCAPG2 is driven by phosphorylated HBO1, which activates H4 histone acetylase, and in turn activates the Wnt/β‐linked protein signaling pathway, promoting GBM cell malignancy and xenograft tumor growth.^196^ In addition, the sirtuins family of deacetylase was also found to be involved in GBM.^197^ In a word, many histone‐modifying enzymes, including methyltransferase, demethylase, acetyltransferase, and deacetylase, overexpression is related to the higher histological grade and poor clinical prognosis of GBM, which needs further clarification in future research. Furthermore, LSD1 and HDAC have a synergistic effect on cell death, which may be a breakthrough in exploring new treatment methods for GBM.

### 3.2 Lung cancer

The number of lung cancer deaths far exceeds that of other cancer types, ranking first in cancer deaths. The results of clinical pathology analysis showed the presence of abnormally modified H3K9me3, H4K16ac, H3K4me2, and H3K9ac in lung cancer tissues.^198^, ^199^ Besides, H4 histone modifications also show abnormal patterns in lung cancer tissues. H4K20me3 is often found in early cancer lesions, and its expression continues to decline as the disease progress.^200^ At the same time, an increase in trimethylation of H3K27 is associated with a longer overall survival and better prognosis.^201^ Therefore, methylation at different sites of histone may cause different clinical prognoses of lung cancer. Moreover, the role of various histone‐modifying enzymes in human lung cancer has also been confirmed. For example, the protein methyltransferase G9a promotes cell invasion and metastasis through the cell adhesion molecule epithelial cell adhesion molecule (Ep‐CAM) in lung cancer.^202^ Besides, Slimane et al. found that SETDB1 is overexpressed in NSCLC and related to cell proliferation and invasion, which is a promising tool for predicting tumor recurrence in early NSCLC patients.^203^, ^204^, ^205^, ^206^ Similarly, many studies have reported the carcinogenic effect of KDM5B. Compared with normal tissues, KDM5B has higher expression in tumor tissues.^207^ KDM5B is associated with malignant proliferation and metastasis of cancer cells, and it has the potential to be a prognostic factor for lung cancer.^208^, ^209^, ^210^ At the epigenetic level, abnormal changes in PTMs directly affect programmed cell death 1 ligand 1(PD‐L1)‐mediated immune resistance,^211^, ^212^ and acetylation of H3K14 and HBO1 participate in the transcription of PD‐L1. HBO1 induces PD‐L1 expression by promoting the enrichment of H3K14ac at the PD‐L1 promoter.^15^ Normally, the PD‐L1 pathway is essential to maintaining immune stability.^213^ However, high levels of PD‐L1 caused by cancers induce T‐cell depletion, enabling tumor cells to evade T‐cell immune attacks.^214^ The molecular compound NBP target at HBO1 to inhibit the acetylation of H3K14, thereby reducing the enrichment of H3K14ac on the PD‐L1 promoters, blocking the PD‐1 and PD‐L1 signaling axis, and inhibiting the activity and proliferation of lung cancer cells.^15^ Recently, remarkable achievements have been made in the immunotherapy of lung cancer. Unfortunately, the effect of immunotherapy is not stable, and some patients still do not respond to immunotherapy. Therefore, the regulation of PD‐L1/PD‐1 axis by targeting HAT HBO1 is of great clinical value. However, the research on the application of NBP in lung cancer immunotherapy is still in the basic research stage. How to develop stable NBP drugs with efficient clinical treatment functions is the focus of future research.

### 3.3 Hepatocellular carcinoma (HCC)

HCC is the most common histological type of liver cancer, and its incidence is expected to exceed one million cases by 2025.^215^, ^216^, ^217^ AFP is an HCC marker that guides cancer treatment and predicts HCC prognosis.^218^, ^219^ Nevertheless, many studies show no significant correlation between AFP level and HCC tumor stage.^220^ Conversely, there is evidence of an interaction between epigenetic changes and HCC.^221^ IHC analysis showed that high expression of H3K4me3 and H3K27me3 indicated poor prognosis and could potentially be a predictive marker of HCC.^222^ The liver characteristics of H3K4me2 and H4R3me2 are related to the late recurrence.^223^ Wong et al. found that HMT G9a was up‐regulated in HCC and accelerated tumor progression by mediating the silencing of tumor suppressor gene RARRES3.^224^ Besides, SETDB1 is an epigenetic enzyme associated with the maintenance of HCC cancer cells that regulates cancer cell growth by p53 methylation.^225^ KDM5B is also overexpressed in HCC samples.^226^, ^227^, ^228^ Knock‐out of KDM5B upregulates P21 and P15 and blocks the cell cycle in the G1/S phase, significantly inhibiting HCC cell proliferation.^227^ It is worth mentioning that histone acetylation also takes part in the progress of HCC. Down‐regulation of MOF of the MYST family significantly reduced the invasion and vascular infiltration of HCC cells.^229^ Recent studies have reported that HBO1 is over‐expressed in HCC cells, compared to normal hepatocytes, resulting in poor overall survival.^230^ Bai et al. found that MiR‐639 is complementary to sequences in the 3′ untranslated regions of HBO1, targeting HBO1 expression and thereby downregulating the HBO1‐mediated Wnt/β‐catenin pathway to inhibit the malignant capacity of HCC cells.^231^ Abnormal phosphorylation modifications of histones H1 and H3 are also associated with HCC.^232^, ^233^ The level of Kcr in HCC has also been found to be related to tumor size and lymph node stage, and its increased expression inhibits the movement and proliferation of cancer cells.^169^ Up to now, the reported histone modifications related to HCC include methylation, acetylation, phosphorylation, and crotonylation. Enzymes that mediate these modifications exert their regulatory effects in various ways, including silencing tumor suppressor genes, promoting P53 methylation, mediating signaling pathways, and regulating cell cycle, which means that we can target its different functions to reverse its malignant biological functions.

### 3.4 Gastric cancer (GC)

Among gastrointestinal tumors, the survival rate of gastric and pancreatic cancers is 5%−20%, most GCs are diagnosed in the late stage because there are no obvious symptoms in the early stage, leading to a high mortality rate.^234^, ^235^, ^236^, ^237^, ^238^ Epigenetic changes are valuable biomarkers for GC prognosis. Jang et al. found that the trimethylated state of H3K9 is highly expressed and positively correlated with tumor staging.^239^, ^240^ SETDB1 is abnormally overexpressed in GC and plays a crucial role in GC occurrence and metastasis by upregulating β‐catenin and matrix metalloproteinase 9.^241^, ^242^ Furthermore, in the xenograft tumor model, the knockout methyltransferase G9a showed a concomitant marked reduction in H3K9 monomethylation and potent inhibition of mechanistic target of rapamycin (mTOR) expression and tumor growth.^243^ Abnormal expression of KDM5B can promote metastasis by regulating various signaling pathways and leading to a poor prognosis. Besides, MiR‐194 can directly target KDM5B and negatively induce GC cell growth.^244^, ^245^, ^246^ In addition, low histone acetylation levels and high expression of HDAC1/2 were also detected in GC tissues.^247^, ^248^ Similarly, histone phosphorylation also plays a vital regulatory role in GC. Ding et al. found that infection of *Helicobacter pylori* (Hp) will cause the increase of H3S10ph, which led to the occurrence of GC.^249^ It is worth mentioning that non‐coding RNA has also been found for the first time to regulate the development of GC through histone acetylation‐modifying enzymes. Jie et al. through RNA‐seq found circMRPS35, which was considered an anti‐oncogene in GC. Furthermore, the RNA pull‐down experiment confirmed that the C2H2 domain of HBO1 (256−315 amino acids) is crucial for its interaction with circMRPS35, which recruits HBO1 into the promoter region of FOXO1/3a and increases the level of H4K5ac to inhibit the progression of GC by upregulating their transcriptional activity.^250^ The study is the first report on the link between circRNA and PTMs, and researchers have established the function of circMRPS35 and HBO1 in inhibiting GC progression. Non‐coding RNA also has an important influence on histone modification of gene promoters. It will be beneficial for further research on the association of non‐coding RNA with PTMs.

### 3.5 Pancreatic cancer

Pancreatic cancer is a fatal disease with increasing incidence worldwide. Chemotherapy is the main method to treat pancreatic cancer in the clinic, but it is easy to produce drug resistance. Therefore, clarifying the molecular mechanism of drug resistance in pancreatic cancer is the key to improve the therapeutic effect of pancreatic cancer. PTMs are important events in the tumorigenesis and development of many other tumors. Therefore, studying PTMs is an important research direction in pancreatic cancer. Dawson et al. concluded through tissue microarray analysis that low levels of H3K4me2, H3K9me2, or H3K18ac portend poor survival for pancreatic cancer.^251^, ^252^ Pancreatic cancer develops resistance to drugs through multiple mechanisms, including dysregulation of key activation signaling pathways and the presence of stromal cells, highly resistant cells, and CSC.^253^, ^254^, ^255^ HBO1 is a substrate of polo‐like kinase 1 (Plk1) and is essential for DNA replication. However, high levels of Plk1 in pancreatic cancer mediate and maintain gemcitabine resistance.^256^, ^257^ Song et al. found that Plk1‐mediated phosphorylation regulates the acetylation activity of HBO1, and HBO1 accumulates in the promoter region of c‐Fos and c‐Jun to constitute the activating protein‐1 (AP‐1) transcription factor that enhances MDR1 expression,^258^ ultimately leading to gemcitabine resistance. Inhibition of Plk1 phosphorylation and its downstream target HBO1 reverse the onset of resistance. However, abnormal expressions of several other histone‐modifying enzymes were also identified.^259^ The high transcriptional levels of HDAC7 and HDAC2 are involved in the progression of pancreatic cancer.^260^ Overexpression of SMYD3 promotes the proliferation, migration, and invasion of pancreatic cancer and acts as a regulator in the cytoplasm by modulating Ras/ERK signaling.^49^, ^261^ In the mouse model, the removal of SETDB1 prevented the formation of pancreatic cancer in mice.^262^ Unfortunately, the reported histone modifications in pancreatic cancer are limited to methylation and acetylation, and other PTMs, such as histone ubiquitination and phosphorylation, have not been reported yet. In the future, the influence of histone modification on pancreatic cancer can be further studied to expand our understanding of histone modification and provide guidance for researching new approaches to treat pancreatic cancer.

### 3.6 Colorectal cancer (CRC)

CRC is one of the most common malignancies in the digestive system, and its mortality rates have been declining for decades owing to advances in screening and improvements in treatment. Understanding the molecular mechanisms underlying CRC is a profound guide for further treatment optimization. There is growing evidence that the pathogenesis of CRC is associated with epigenetic modifications.^263^, ^264^, ^265^ The high levels of H3K9me3 and H4K20me3 are related to the better prognosis of CRC, as well as the low nuclear expression of H3K4me3.^266^, ^267^, ^268^ Multiple studies have demonstrated that the methyltransferase SETDB1 is up‐regulated in CRC and is associated with higher histological grades and Tumor‐Node‐Metastasis (TNM) staging.^269^, ^270^ Furthermore, the cell cycle was blocked in G1 phase after the silence of SETDB1.^270^ Significant expression of another methyltransferase, PRMT1 was also identified in CRC patients.^271^, ^272^ JMJD2D specifically demethylates H3K9me2/3, which also contributes to the progression of CRC.^273^ Two other demethylases, KDM5B and LSD1 were also found to promote the distant metastasis of CRC cells.^274^, ^275^, ^276^, ^277^

Histone acetylation modifications also play an important regulatory role. Kuppen et al. reported that high acetylation levels of H3K56 and H4K16 were highly correlated with higher survival in CRC patients.^278^ HDAC2/3 expression was significantly increased in CRC, suggesting that HDAC2/3 may have a meaningful effect on the progression of CRC.^279^, ^280^ Taniue et al. found that Wnt/c‐Myc signaling is involved in the inhibition of H3K14 acetylation via the HBO1 complexes, leading to the downregulation of tumor suppressor candidate 3 (TUSC3), thus promoting the proliferation of CRC cells.^281^ Therefore, under the cellular background of CRC, high levels of H3K9me3, H4K20me3, H3K56ac, and H4K16ac predict a better prognosis for patients. Similarly, low trimethylation levels of H3K4 and acetylation of H3K14 may be related to a better prognosis. The methylation and acetylation modification patterns that lead to entirely different results are still unclear. Each site of histone modification has its unique molecular regulation mechanism, which should be further explained in the future.

### 3.7 Breast cancer

Breast cancer is one of the leading causes of death in women. Breast cancer alone accounts for 30% of all new diagnoses in 2021, and its incidence now exceeds that of lung cancer.^282^ To improve the treatment methods for breast cancer, it is critical to identify useful biomarkers of breast cancer that respond best to treatment. However, hormone therapy with ERa as the main body makes tumors prone to develop therapeutic drug resistance, more than 9 million breast cancer survivors worldwide suffer from menopausal estrogen consumption‐related symptoms and the side effects of cancer treatment.^283^ Recent studies show that epigenetics plays a key role in the occurrence and development of breast cancer.^284^, ^285^, ^286^ Cancer tissues exhibit intercellular differences in the total number of specific histone modifications.^287^ Low or absent levels of H4K16ac are found in most breast cancer cases (78.9%), which are early signs of breast cancer.^288^, ^289^ During the EMT, the loss of H4K16ac in mesenchymal cells can be used as a marker to distinguish between epithelial and mesenchymal phenotypes.^284^ Wang et al. noted that HBO1 and ER have multiple mutual regulatory effects. 17b‐estradiol (17b‐E2) upregulates the levels of HBO1 via the ERK1/2 pathway, and HBO1 is positively related to the histological grade of ERα‐positive breast cancer.^290^ In addition, HBO1 is involved in the ubiquitination of ERα in vivo and, thus, leads to its destabilization.^14^ Interactions were also found between ERα and p300 of the HATs family. P300 is recruited to ERα by the steroid receptor co‐regulatory factor (SRC), which forms a transcriptionally active ERα/SRC‐3/p300 complex.^291^, ^292^ Moreover, the intrinsic acetyltransferase activity of p300 can acetylate ERα, which enhances the DNA‐binding activity of ERα.^293^

Among histone methylation modifications, low levels of arginine (H4R3me2) and lysine methylation (H3K4me2 and H4K20me3) are associated with poor prognosis.^289^ Conversely, high expression of H3K4me3 and H3K9ac are related to low survival rates of breast cancer.^294^ Methyltransferase SETDB1 regulates the progression of breast cancer and its chemotherapy resistance through complex interactions with various molecules.^295^, ^296^ C‐MYC could enhance the transcription of SETDB1, making SETDB1 a potential mechanism for driving breast cancer progression.^296^ Liu et al. also found that E2H2 is abnormally expressed in breast cancer and has the potential to be a therapeutic target.^297^ Moreover, Chen et al. reported the competence of LSD1 to reduce the expression of tumor suppressor genes by interacting with β‐catenin.^298^, ^299^ In addition, the down‐regulation of KDM5B in TNBC inhibits long non‐coding RNA MALAT1, thereby inhibiting cancer cell invasion.^300^, ^301^ One thing we should pay attention to in the above research is that dimethylation and trimethylation at the H3K4 site have opposite clinical significance for the prognosis of breast cancer. It means that histone methylation modification has a very strict and delicate regulation system. Compared with acetylation and ubiquitination, methylation modification has various forms and more complex functions, which will have significant research value in the future.

### 3.8 Ovarian cancer (OC)

OC is the main cause of death in women with reproductive malignant tumors. The initial operation, with the goal of R0 resection and platinum‐based combination chemotherapy, is the standard treatment for OC.^302^, ^303^ Although the initial pharmacotherapy was encouraging, long‐term use of drugs resulted in drug resistance and was one of the main reasons for the discontinuation of cisplatin during chemotherapy.^304^, ^305^, ^306^ Xie et al. showed that the application of epigenetic approaches for the diagnosis, clinical treatment, and prognosis of OC is a promising area for future clinical research.^307^, ^308^, ^309^, ^310^ New evidence suggests that epigenetic changes have important implications in OC drug resistance.^311^, ^312^ Abbosh et al. found that inhibiting H3K27 methylation re‐sensitizes drug‐resistant OC cells to cisplatin.^313^ Moreover, loss of H3K27me3 can increase the expression of tumor suppressor genes, thereby leading to the occurrence of tumors.^313^, ^314^ LSD1 is overexpressed in OC, and its interaction with H3K4 leads to the down‐regulation of E‐cadherin, thus enhancing cell migration and invasion capacity.^315^ It can also regulate the autophagy of OC cells through AKT/mTOR signaling pathway.^316^ KDM2B is highly expressed in OC and upregulates the level of the EZH2 gene, and they both play an important role in the tumorigenesis of OC.^317^, ^318^ Quintela et al. also identified that HBO1 is highly upregulated in OC.^319^ HBO1 participates in histone H4 acetylation mainly through JADE2 and subsequently regulates YAP1 expression to affect the mechanical phenotype of OC cells.^319^ Up to now, all reports have shown that histone modification patterns have malignant potential in OC and participate in the development of OC drug resistance. However, there are rare studies about PTM in OC at present, and the role of PTM in OC remains unclear, which needs further research.

### 3.9 Osteosarcoma (OS)

OS is the most common bone malignancy. However, its survival rate has only minimally improved over the past 30 years.^320^, ^321^, ^322^ Therefore, new therapeutic methods are needed to improve the therapeutic efficiency of patients with refractory OS. In vitro studies have shown that HBO1 silencing or knockdown can effectively inhibit cell viability and proliferation, and the overexpression of HBO1 in OS tissues is associated with poor overall survival.^323^ ZNF384 can directly promote the transcription of HBO1, leading to a malignant phenotype in OS cells.^323^ WM‐3835 is an effective HBO1 inhibitor that can directly inhibit cell viability and proliferation while inducing apoptosis.^323^ Moreover, SETDB1 has also been confirmed to be related to the pathophysiology of OS.^324^ However, no report expounds on the carcinogenic mechanism of SETDB1 in OS until now. There is little research on histone modification in OS, which weakens potential treatment methods for OS treatment. Currently, only two histone‐modifying enzymes are related to OS, so exploring more enzymes with potential mechanisms in OS is of great guiding significance.

### 3.10 Acute myeloid leukemia (AML)

**FIGURE 4:**
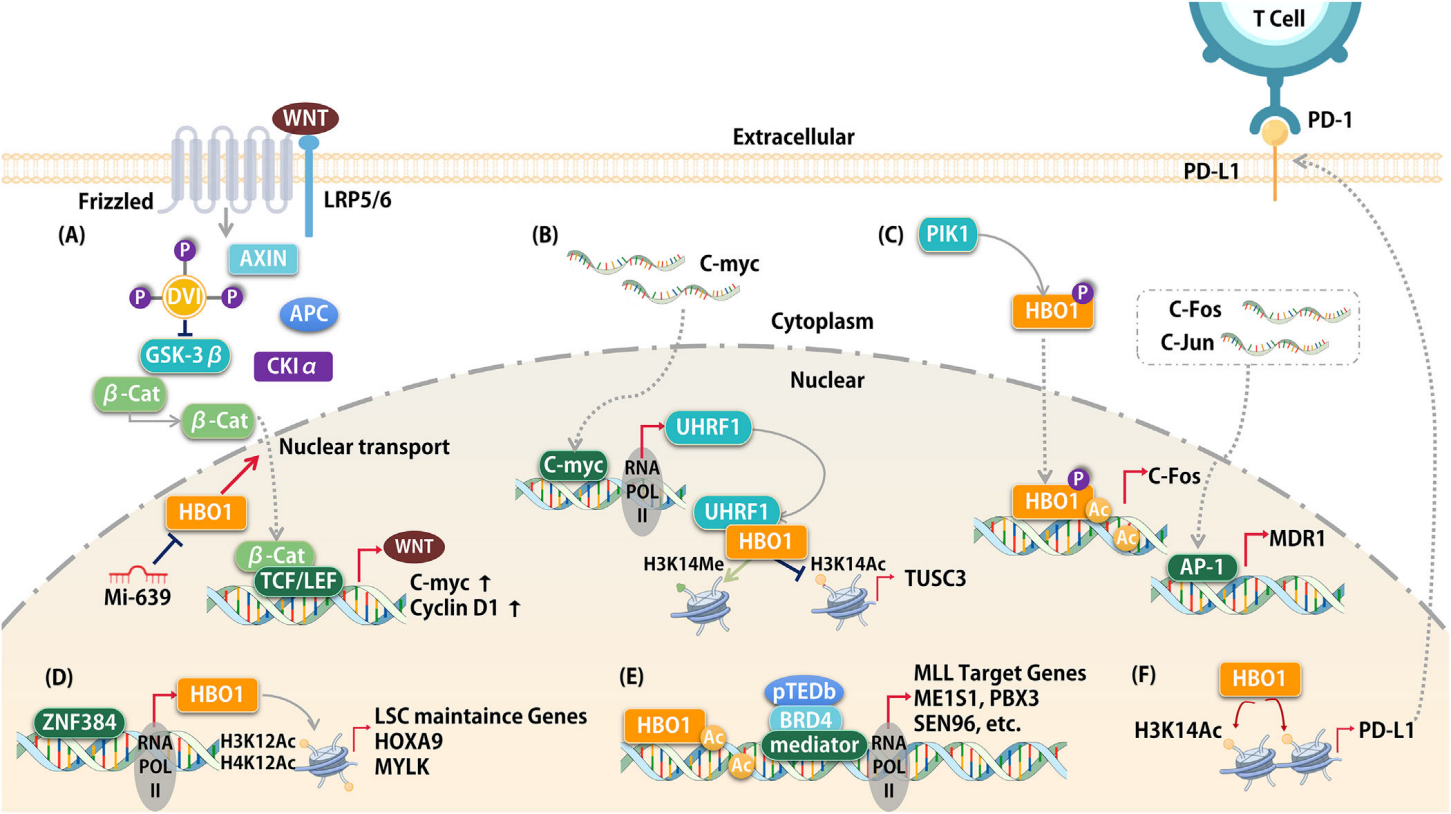
Many complex molecular interactions of HBO1 are involved in tumorigenesis. (A) miR‐639 inhibits HBO1‐mediated translocation of β‐linked proteins from the cytoplasm to the nucleus. (B) UHRF1 partially inhibits KAT7‐mediated acetylation of H3K14. (C) Polo‐like kinase 1 (Plk1) phosphorylation of HBO1 on elevated levels of c‐Fos and MDR1. (D) ZNF384 is an important transcription factor that binds directly to the HBO1 promoter. (E) HBO1‐mediated histone acetylation may serve as a scaffold for BRD4 binding to chromatin. (F) HBO1 binds to the PD‐L1 promoter and epigenetically induces PD‐L1 expression.

AML is a hematologic malignancy, characterized by abnormal gene activation, which causes uncontrolled self‐renewal of hematopoietic progenitors.^325^, ^326^, ^327^ The leukemic state is maintained by LSC, which have a high capacity for proliferation and self‐renewing. The inability to destroy LSC results in the limited therapeutic efficacy of AML.^328^, ^329^, ^330^ A better understanding of AML pathogenesis at the molecular level is important for guiding more precise treatment decisions in the future. The prospect of epigenetics in the research and treatment of leukemia has gradually gained focus.^331^, ^332^, ^333^, ^334^ The genetic heterogeneity of leukemia poses a therapeutic challenge. However, drugs targeting epigenetic mechanisms components are expected to be an integral part of leukemia treatment.^327^, ^335^, ^336^ The BRPF2 subunit regulates the HAT activity of HBO1.^337^, ^338^ The BRPF2‐HBO1 complex is co‐located in the genome, and regulation of its HAT activity to introduce H3K14ac is necessary for erythropoiesis in the fetal liver.^339^ In AML, the HAT structural domain of HBO1 mediates H3K14ac and maintains high expression of HOXA9 and HOXA10, which are critical genes for the functional properties of LSC.^340^ Yan et al. found that HBO1‐mediated histone acetylation provides a platform forMixed Lineage Leukemia (MLL) fusion‐associated linker proteins (e.g., BRD4 and AF4) to recruit gene promoters to maintain the expression of critical genes involved in MLL‐AF9 propagation^341^ (Figure 4). In chronic myeloid monocytic leukemia (CMML), NUP98‐HBO1‐derived oncogenic features are regulated by histone acetylation, and H4 and H3 significantly activate oncogenic HOXA9 features, leading to CMML development.^341^, ^342^ Although these studies have pointed out that HBO1 has a cancer‐promoting effect, HBO1 can also exert anti‐cancer effects in AML. Sauera et al. showed that HBO1 acts as a growth inhibitor and that its primary target, H4K5, is inhibited in leukemia and associated with poor overall survival^343^ (Table 2). Preliminary clinical trials have also found that HDACIs are effective in AML, causing silenced oncogenes to be re‐expressed in cancer cells.^344^ The changes produced by epigenetic alterations act differently in different cellular backgrounds, resulting in different expression levels of HBO1 as an oncogene or a tumor suppressor gene in different cell lines. Therefore, the study of its expression and function in cancers with different genetic backgrounds is important and needs to be clarified in the future.

**TABLE 2:** Functional characterization and clinical significance of HBO1 in various tumors.

**Cancer type**	**Expression**	**Functional role**	**Related genes/pathway**	**Relevant clinicopathological features**	**Clinical value**	**Ref**.
GBM	Overexpression	Cell proliferation, migration, and invasion, cell cycle	NCAPG2, Wnt/β‐catenin signaling, Cyclin E, Cyclin D1, MCM2, MCM6	/	/	^196^
Lung cancer	Overexpression	Cell viability, proliferation, colony formation	NBP, RNA pol II, PD‐L1	/	/	^26^
HCC	Overexpression	Cell viability, proliferation, migration and invasion, colony formation, apoptosis, cell cycle, tumor volume, EMT	Wnt/β‐catenin signaling, Mi‐639, caspase‐3, caspase‐9, MYLK, VEGFR2, PBX3, CCR2, HOXA10, FRZB	Overall survival, tumor volume	Therapeutic target	^230^, ^231^
GC	Overexpression downexpression	/	CircMRPS35, FOXO1, FOXO3a, P21, P27, E‐cadherin, TWIST	Invasive depth, tumor differentiation, lymph node and distant metastasis, TNM staging, CEA level	Prognosis biomarker	^239^, ^250^
Pancreatic cancer	Overexpression	/	Plk1, c‐Fos, MDR1	/	/	^258^
CRC	Downexpression	/	Wnt/β‐catenin signaling, C‐Myc, UHRF1, TUSC3	/	/	^281^
Breast cancer	Overexpression	CSC formation, EMT, colony formation	E2‐ERK1/2 signaling pathway, LMW‐E/CDK2, ERa, ALDH, VIM, TWIST, SLUG, N‐cadherin, E‐cadherin	Overall survival, ERα and PR expression, histology grade	Therapeutic target, diagnosis biomarker	^25^, ^285^, ^286^, ^290^
OC	Overexpression	Cell viability, cytoskeletal organization and microtubule dynamics	YBX1	/	/	^319^
Bladder cancer	Overexpression	Proliferation, invasion, colony formation, cell cycle, invasion, tumor size	Wnt/β‐catenin signaling, Cyclin D1	T classifications, overall survival, recurrence‐free survival, tumor stage, tumor size	Therapeutic target, prognostic biomarker,	^382^
OS	Overexpression	Cell viability, proliferation, migration and invasion, apoptosis, cell cycle, mitochondrial apoptosis, tumor volume	WM3835, ZNF384, HOXA9, caspase‐3, caspase‐9, PARP, MYLK	Overall survival	Therapeutic target	^323^
Leukemia	Overexpression downexpression	Proliferation, apoptosis, cell cycle, colony formation	MML‐AF9, NUP98, BRD4, Hoxa9, Hoxa10, Irf8	Overall survival	Therapeutic target	^340^, ^341^, ^342^, ^343^

Similar to the unique anti‐cancer mechanism of HBO1 in AML, SETDB1 shows a lower expression level in AML, while the higher level of SETDB1 positively correlates with more favorable overall survival, and its mediated H3K9me3 has become an important epigenomic marker of AML.^345^, ^346^ Stefanie et al. found that low EZH2 protein levels and their mediated reduction of H3K27me3 levels confer resistance to AML and are associated with poor patient outcomes.^347^ In addition, the activity of PRMT1 and PRMT5 enhances the progress of AML in vitro and vivo.^348^, ^349^, ^350^ LSD1, an important regulator of the heme synthesis pathway,^338^ is also frequently overexpressed in AML.^351^, ^352^ Methylation modification and acetylation modification of histones have been studied in depth in AML. However, some modifications of histones, such as propionylation and crotonylation, still remain unclear on cancer pathophysiology.

## 4 THERAPEUTIC ENZYME TARGETS IN PTMS OF HISTONES

Many abnormal histone modification sites and the activity changes of histone modification enzymes in cancer have been described above. Different enzymes have the function of catalyzing the modification of different histone sites. Therefore, targeting these histone‐modifying enzymes with carcinogenic potential is an important method for cancer treatment. At present, the studies on inhibitors of HMT, HDM, HAT, and HDAC are the global research hotspot. These enzyme inhibitors can alter the levels of histone modifications in specific regions of chromatin, thereby affecting gene expression. It has become a new class of anti‐tumor drugs with broad development prospects and application value nowadays.

### 4.1 HMT inhibitors

**FIGURE 5:**
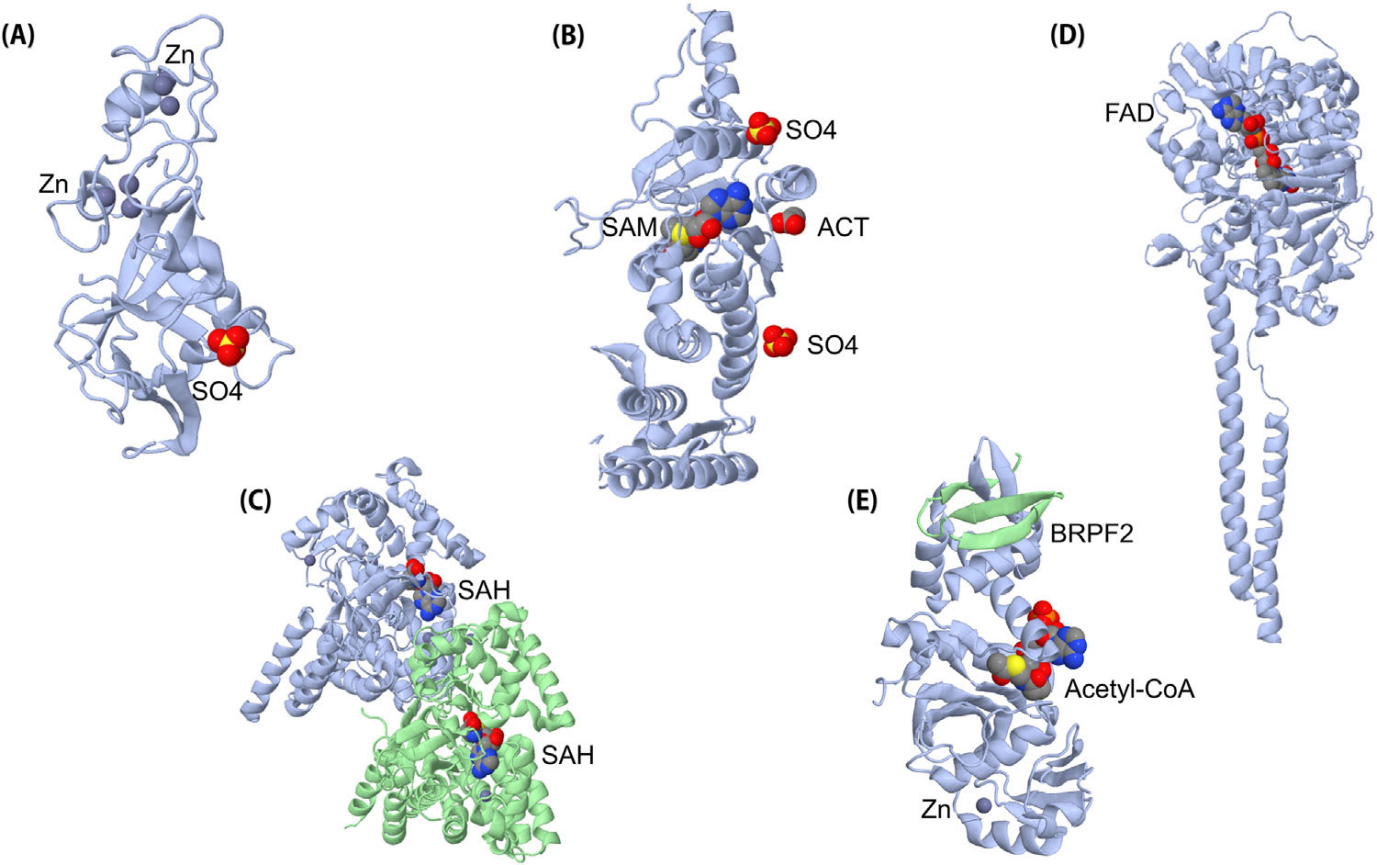
The tertiary structure and corresponding ligands of several common histone transferases. (A–C) The tertiary structures of three histone lysine methyltransferases. (A: EZH2, PDB number 4MI5; B: DOT1, PDB number 1NW3; C: SMYD3, PDB number 3OXF). (D) The tertiary structure of histone lysine demethylase LSD1 (PDB: 2DW4). (E) Cartoon model showing the tertiary structure of HBO1 (PDB: 5GK9), whose subunit acetyl‐CoA regulates acetyltransferase activity and protein stability. BRPF2 is specifically bound to the C‐terminal of HBO1 with a hairpin structure. The formation of the complex can promote the activation of histone H3K14 acetylation by HBO1 in vitro.

Inhibitors of HMT have developed rapidly in the past decade. The genetic changes of HMT have induced the initiation of human diseases. The abnormal expression patterns of EZH2, DOT1, SMYD3, and SETDB1 have been found in many cancers, such as breast cancer, OC, HCC, and NSCLC.^324^, ^353^, ^354^ Their unique structure and biological functions in tumors make them potential therapeutic targets for tumors (Figure 5). EZH2 is the subunit of the catalytic core of PRC2 in polycomb‐group proteins family, and its high levels have been found in many human cancers including bladder cancer, NSCLC, and CRC.^355^, ^356^ As a new anti‐tumor drug in the epigenetics field, the development of EZH2 inhibitors is at the forefront, and one drug has been listed. Tazemetostat was approved by the FDA in 2020 for the treatment of follicular lymphoma and metastatic/locally advanced epithelioid sarcoma, which is not suitable for complete resection.^357^ In addition, many kinds of EZH2 inhibitors are undergoing clinical studies, such as GSK126, EZH1/2 dual inhibitor valemetostat, CPI‐1205, and so forth.^355^, ^358^, ^359^ Among them, Chen et al. reversed EZH2‐mediated H3K27me3 using the small molecule compound EPZ011989, thereby inhibiting SCLC tumor growth.^360^ Another methyltransferase inhibitor, SMYD3, has also been clinically tested in several diseases and studied in depth, especially in cancer. BCI‐121 is also a small molecular compound that can induce a significant decrease in SMYD3 activity in CRC cells in vitro, where BCI‐121 plays an anti‐OC cell proliferation role by causing S‐phase arrest and increasing the rate of apoptosis.^361^, ^362^ Additional competitive inhibitors of SMYD3 include GSK2807, EPZ031686, EPZ030456, and BAY‐6035.^363^, ^364^, ^365^ However, there is no effective inhibitor of SMYD3. DOT1L is also an HMT, and clinical trials using the DOT1L inhibitor EPZ‐5676 for hematological malignancies are also in progress.^366^

Notably, researchers have found that inhibition of SETDB1 can make immunotherapy more effective for patients, and the protein is essential for the study of new anti‐cancer drugs.^367^ It is worth noting that the approved chemotherapy drug paclitaxel has been verified to affect SETDB1 levels.^368^ SETDB1 could catalyze the methylation of histone H3K9, and excessive H3K9 trimethylation products promote the inhibition of tumor suppressor factors, leading to tumor occurrence. SETDB1 has a tandem Tudor domain (TTD) that recognizes sequences containing histone H3. SETDB1‐TTD‐IN‐1 is a selective inhibitor of SETDB1‐TTD.^369^ At present, no specific inhibitor of SETDB1 has been found. The SET domain of SETDB1 is split, so it is more difficult to develop specific inhibitors of SETDB1. Therefore, the development of inhibitors targeting other domains of SETDB1, such as inhibitors of the SETDB1‐TTD domain, is of great significance to treat diseases associated with abnormal expression of SETDB1. Regrettably, the research progress on these abnormally modified enzymes is relatively backward, compared with the widespread distribution of HMT in cancer. Nowadays, only tazemetostat has been approved for marketing, and most of the other HMT inhibitors are still in the basic research stage, which has great clinical potential. It is the focus of future research to optimize the efficacy of these inhibitors and reduce their toxic and side effects.

### 4.2 HDM inhibitors

Histone methylation was once considered irreversible until the discovery of LSD1 confirmed the existence of HDM, which provided new sight for the mechanism of histone modification and the corresponding drug research. LSD1 can specifically remove the monomethylated and dimethylated groups at the H3K4 and H3K9 sites.^44^ Researchers found that LSD1 dysfunction may play a key role in a variety of cancers, and inhibition of LSD1 leads to tumor stem cell maintenance disorder and inhibition of tumor growth.^370^ Inhibitors of LSD1 can re‐express these abnormally suppressed genes (SFRP1, SFRP4, SFRP5, and GATA5) in CRC cells, thus inducing cell apoptosis.^371^ At present, a variety of LSD1 inhibitors have entered clinical research, including GSK2879552, tranylcypromine (TCP), GSK354, and GSK690.^372^ The inhibitor of monoamine oxidase, TCP can inhibit LSD1, but its selectivity is barely satisfactory. GSK2879552, a selective irreversible LSD1 inhibitor, is in clinical trials in the United States. It is used to treat recurrent and refractory SCLC.^372^, ^373^ Interestingly, 4SC‐202, a small molecule compound, has dual functions of inhibiting HDAC and LSD1 and is currently used in clinical trials for malignant melanoma and advanced hematological malignancies patients.^374^, ^375^ However, the development of the JMJC family of protein inhibitors that also contain demethylase activity has been difficult. In the research by Zhang et al., it was found that GSKJ4 exerted an inhibitory effect on breast cancer stem cells by inhibiting the activities of histone demethylation metastasis KDM6A (UTX) and KDM6B (JMJD3), with the potential to be used as a targeted therapy for breast cancer.^376^ GSKJ4 has a weak inhibitory ability on KDM5B, while KDM5B is also abnormally expressed in a variety of tumors, such as GBM and breast cancer.^195^, ^300^, ^377^ More recently, a KDM5B inhibitor, AS‐8351, has been demonstrated to inhibit the proliferation ability of breast cancer cell.^378^ Compared with HMT inhibitors, the development of HDM inhibitors is less advanced. The difficulty in developing JMJC inhibitors seems to have limited the development of HDM inhibitors. Currently, there are no approved HDM inhibitors on the market. Fortunately, HDM inhibitors are mainly in the clinical research stage. The main goal in the future is to clarify the target indications of experimental drugs and determine the best therapeutic regimens of new medicines. Furthermore, the development of JMJC inhibitors will be the key research direction in the future.

### 4.3 HAT inhibitors

Due to the balancing function of histone acetylation and deacetylation in multiple life activities, a great diversity of HDACs and HAT inhibitors have been developed so far. Among the existing small molecule inhibitors of HAT, there are many studies targeting p300/CBP inhibitors. Based on a virtual screening using the crystal structure of p300 HAT/Lys‐CoA, C646, and A‐485 are the two most representative inhibitors of p300/CBP. However, their effectiveness in clinical treatment needs to be further determined in future studies.^379^, ^380^, ^381^ Besides, WM‐3835 exerts a selective inhibitory effect on HBO1 of the HAT family.^382^ WM‐3835 has been proven effective in inhibiting the growth of mouse OS xenografts. The small‐molecule NBP inhibits PD‐L1 expression by targeting HBO1 to mitigate lung cancer progression.^15^ Compared with the tremendous clinical success of HDACs, the research on HATs needs to catch up. Only two drugs targeting HBO1 have been reported in the preliminary basic research stage. Studies on other types of HATs, such as p300/CBP inhibitors, are still in the initial stage, and there is no basic research on whether the predicted drugs have clinical therapeutic value. Therefore, it is a valuable research direction to verify the effectiveness of these drugs, which can enrich the types of HAT inhibitors as well.

### 4.4 HDAC inhibitors

**FIGURE 6:**
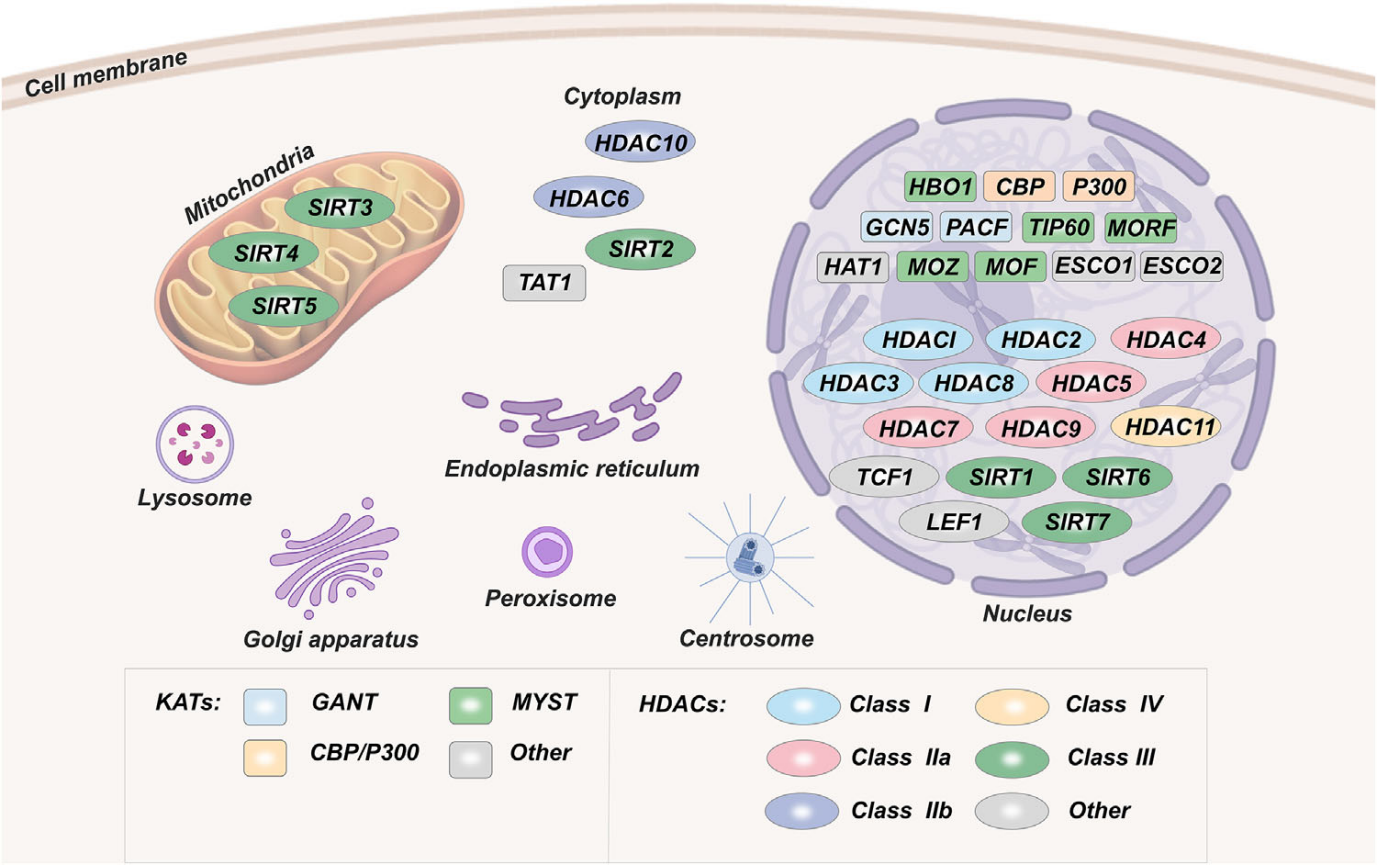
HAT and HDAC are localized in the cytoplasm, nucleus, and mitochondria of mammalian cells.

Overexpressed HDACs found in many cancers are closely related to poor clinical prognosis, making them popular targets for studying different cancers. HDACIs, are the most advanced drugs aimed at epigenetics. HDACs can be classified into classical zinc^2+^‐dependent HDACs and NAD‐dependent sirtuin deacetylases.^55^, ^157^ HDACs can be further classified into Classes I, IIa, IIb, III, and IV, based on the homology of yeast proteins^383^, ^384^ (Figure 6). Inhibition of HDAC enables global acetylation (e.g., H3K9ac, H3K18ac, H3K23ac, H3K56ac, H4K5ac, H4K8ac, and H4K16ac).^71^ Each class can be inhibited to varying degrees by the existing HDACIs. HDACIs can reactivate tumor suppressor factors by eliminating abnormal acetylation states in cancer cells.^385^, ^386^ Extensive research has been conducted and results have been obtained on the function, activity regulation, and structure‐guided drug design of HDACIs. Previous clinical trials have demonstrated that HDACIs can significantly inhibit tumor growth by inducing cell division defects and intrinsic apoptosis. Now a few HDACIs have been clinically used for cancer treatment.^387^, ^388^ Vorinostat (SAHA) and romidepsin are non‐specific inhibitors of a wide range of HDACIs and have been approved by the FDA for treating cutaneous T‐cell lymphoma.^389^, ^390^, ^391^ Besides, the FDA approved belinostat in 2014 as a monotherapy for relapsed or refractory peripheral T‐cell lymphoma.^392^ As a listed therapeutic drug for peripheral T‐cell lymphoma, chidamide is also included. Chidamide combined with chemotherapy can improve the median progression‐free survival of patients, and it is the first approved oral subtype selective HDACI in China.^393^, ^394^ HDACIs have also been found to be effective in treating multiple myeloma. The combination of panobinostat with bortezomib and dexamethasone was approved by the FDA in 2015 for patients who had received at least two treatment programs before.^395^, ^396^

The indications of HDACIs currently are mostly limited to the peripheral T‐cell lymphoma and cutaneous T‐cell lymphoma market, and they have little effect on treating solid tumors. So far, no reasonable medication strategy has been approved and marketed for solid tumors. However, SAHA was found to have a sensitizing effect in the radiotherapy of NSCLC. SAHA significantly sensitizes the tumor cell lines to the radiation effect of 2 Gy irradiation, which induces the hyperacetylation of H4.^397^ In addition, Rivera et al. found that abexinostat had a similar anti‐tumor effect as SAHA in enhancing radiotherapy for NSCLC.^398^ A second‐generation HDACI, quisinostat, has also been involved in an effective treatment method for NSCLC. Bao et al. revealed that quisinostat increases p53 acetylation at the K382/K373 sites upregulates p21 expression and leads to the G1 phase stagnation.^399^ Besides, a clinical study named SHELTER confirmed that the combined use of resminostat and sorafenib has a specific therapeutic effect and may have further research value in HCC.^400^ In addition to selectively mediating cytotoxicity, HDACI is also capable of inducing immune changes. Entinostat, a class I HDAC selective inhibitor, increases the infiltration of effector T cells and MHC‐II expression, to enhance the anti‐PD‐1 and anti‐tumor effects of radiation.^401^

HDACIs are potential proliferation‐inhibiting compounds, with dozens of HDACIs at various stages of development. There are many subtypes of HDACIs with different tissue distribution and physiological functions. However, most of the current HDACIs are broad‐spectrum inhibitors, but a precision medicine perspective is essential for the future development of epigenetic therapies. Therefore, developing more effective inhibitors with cell selectivity and subtype specificity for HDACs is a great challenge. Besides, at present, the clinical indications of HDACI treatment are limited to hematological tumors, and the research and development of drugs for solid tumors are relatively backward. Currently, the potential HDACI drugs for solid tumors are still in the basic research stage. Therefore, combining HDACIs with chemoradiotherapy or with drugs such as immune checkpoint PD‐1/PD‐L1 has become an important future direction. In the future, a large number of basic studies can be carried out in the pharmaceutical field for the indication expansion of HDACIs.

## 5 CONCLUSION AND FUTURE DIRECTIONS

The complexity and research difficulty of PTMs are greater than the linear relationship between genes and traits involved in traditional DNA. However, with the deepening understanding of PTMs, more and more scientists have begun to face up to the biological value of histones. As an essential epigenetic marker, histone modification is also related to other epigenetic markers to some extent, and they form a complex network together. Histone codes greatly enrich the information of traditional genetic codes. The diverse modifications of histone amino‐terminal expand the information base of genetic code. Over the past two decades, significant advances have been made in identifying histone PTMs. Changes in the epigenetic landscape of cancers can affect the expression of genes involved in cellular metabolism, primarily through aberrant DNA methylation, histone modifications, and dysregulation of metabolic signaling pathways by non‐coding RNA.^402^, ^403^ Although the mechanisms that drive tumorigenesis are still not fully understood, the integration of protein PTMs significantly increases our understanding of this larger picture. In this review, we investigated plentiful functions of PTMs and how they regulate key proteins involved in several important cancer‐related cellular events. Notably, these functions include DNA damage and repair, aging process, tumorigenesis, and CSC development. In the cancer background, most of the histone‐modified enzymes work as a carcinogenic factor, but there are also exceptions, such as HBO1 of the HAT family can inhibit cancer in GC, CRC, and AML. This may be due to the heterogeneity of different cell lines. Thus, the change from epigenetic changes inevitably depends on the cancer background and the cells. In addition, the histone modification crosstalk between histone acetylation and other PTMs is interesting. For example, methylation of H3K4 can enhance the acetylation activity of HAT at the H3 tail. Crosstalk between histone modifications is not limited to acetylation or methylation. Research confirmed that H3K14 not only exists as a star histone acetylation site but also as a site of Kpr and Kbu in vivo. This interesting cross‐functional modification of histones is expected to become a hot topic for future research.

With the continuous development of the basic theory of epigenetics and related research, epigenetic drugs have become a newly rising field. Epigenetic therapy is devoted to promoting the normalization of PTMs with malignant phenotypes, and its regulation involves the mechanism of DNA replication, DNA repair, and RNA translation. Fortunately, chromatin‐mediated genomic regulation is amenable to use as demonstrated by the many successful epigenetic therapies. Therefore, PTM‐based therapy appears to be a candidate drug for reducing the burden of cancer and metabolism‐related diseases. Until now, targeted drugs by PTMs can be divided into four categories: HMT, HDM, HAT, and HDACIs. The HMT inhibitor tazemetostat and five HDACIs, vorinostat, romidepsin, belinostat, chidamide, and panobinostat, have been approved for marketing in recent years. Many of the remaining drugs are still at various stages of development. Most HMT inhibitors are in the primary research stage, except the representative tazemetostat, and it is very promising to select suitable drugs from them to enter clinical trials. The development of acetyltransferase inhibitors, however, is very limited. At present, only HBO1 inhibitors have been reported and are in the in vitro research stage. Other potentially effective compounds of HAT, such as the p300/CBP family, have not been confirmed by research yet. It is still worth exploring the field to develop drugs for HAT inhibitors in the future. The study of HDM and HDACIs is the most promising drugs for clinical use in the near future, and most of them are at different stages of clinical trials. Especially, HDAC has developed very rapidly. Presently, five kinds of drugs of HDACIs have been widely used in clinic. However, the listed drugs are still limited to the treatment of hematological tumors, such as T‐cell lymphoma and multiple myeloma. At the same time, other HDACIs are in the initial research stage of solid tumor, which are very promising to further broaden the scope of clinical application of HDACIs and add new methods to epigenetic therapy. Future development of more complete system models, including their combined functions and mechanisms, will be critical for the flexible use of this method to treat diseases. The relationship between histone modifications and disease states has aroused the interest of many researchers, and understanding the interactions between epigenetic changes helps to identify mutations that contribute to vulnerability and can be used as therapeutic targets. Therefore, establishing a perfect and accurate detection system and developing and designing truly effective inhibitors are current problems that must be solved. Only when the specific mechanism of action of PTMs on tumors is clarified, it is possible to conduct targeted research on anti‐tumor drugs targeting PTMs according to their molecular mechanism and promote cancer treatments.

This review reveals the balance of PTM in the human body. It emphasizes that further research is needed to understand the molecular function of PTMs in cancer pathogenesis and its relationship with cancer prognosis. We believe that the following points are worthy of being studied in the future: (i) methods of developing sensitive and specific means for detecting novel histone PTMs; (ii) methods to explore and expand the form of histone modification crosstalk; (iii) the molecular biological significance of numerous histone PTMs and the mechanism by which they are involved in the regulation of normal and abnormal life processes; and (iv) since there are many types of histone modifications, drugs potentially acting on PTMs need to be explored for the treatment of diseases caused by abnormal regulation of histone PTMs.

## AUTHOR CONTRIBUTIONS

R.L. wrote the original draft. J.W. and H.G. investigated and wrote the original draft. W.Y. curated data and visualized the study. S.L conceptualized the study. Y.L. wrote the manuscript, as well as reviewed and edited the manuscript. Y.J. did the investigation for the manuscript. X.L. supervised and managed project administration. H.Z. conceptualized and acquired funding. J.T. supervised the study. All the authors have read and approved the final manuscript.

## CONFLICT OF INTEREST STATEMENT

The authors declare that they have no competing interests.

## ETHICS STATEMENT

The authors declare that ethics approval was not needed for this study.

## Data Availability

Not applicable.
